# Cystinuria Diagnosed at Age 67 Following a 49‐Year Diagnostic Delay: A Reminder of Indolent Cystinuria in Adult Urolithiasis—A Case Report

**DOI:** 10.1155/criu/8475574

**Published:** 2026-09-19

**Authors:** Sarah Jeffery, Matthew Tyson, Laura McAuley, John Harty

**Affiliations:** ^1^ Urology, Craigavon Area Hospital, Southern Health & Social Care Trust, Craigavon, UK, hscni.net; ^2^ Urology, Bon Secours Hospital, Cork, Ireland, bonsecours.com; ^3^ Nephrology, Daisy Hill Hospital, Southern Health & Social Care Trust, Newry, UK, southerntrust.hscni.net

**Keywords:** case report, cystinuria, kidney calculi, urolithiasis, urology

## Abstract

Cystinuria is a genetic disorder resulting from mutations in the SLC3A1 and SLC7A9 genes, which impair the reabsorption of cystine, ornithine, lysine and arginine and lead to increased urinary excretion of cystine. Elevated urinary cystine concentration promotes precipitation and subsequent stone formation, manifesting as recurrent urolithiasis. Cystinuria is the most common genetic condition that results in urolithiasis and is typically diagnosed in adolescence. In this case report, we present a male patient diagnosed with cystinuria at the age of 67. The patient presented with acute left flank pain, and noncontrast CT imaging of the urinary tract identified left renal calculi and multiple proximal ureteric stones, accompanied by hydronephrosis. Ureteroscopy revealed dense calculi resistant to laser fragmentation, and stone analysis confirmed a 100% cystine composition. The patient reported a previous stone event at age 18 with spontaneous passage but had remained stone‐free for nearly 50 years and had no prior diagnosis of cystinuria. This case demonstrates that cystinuria, although typically diagnosed in childhood or adolescence, can be diagnosed in older adults with recurrent or resistant urolithiasis. Clinicians should maintain a high index of suspicion for cystinuria in adult patients with unexplained or refractory stone disease, and stone analysis remains essential for accurate diagnosis. Optimal management centres on preventing recurrent stone formation through aggressive fluid intake, dietary modifications and medical alkalinisation of urine. Multidisciplinary follow‐up is important to reduce the risk of renal impairment and potential later need for nephrectomy.

## 1. Introduction

In this case report, we present a male patient diagnosed with cystinuria at the age of 67, after a 49‐year diagnostic delay. Urolithiasis is a common condition with a prevalence of 5%–10% in European adults [1]. Aetiology includes environmental and genetic factors, with heritability found to be > 45% in twin studies [2]. Fifteen percent of patients attending stone clinics (11.4% of adult patients and 20.8% of paediatric patients) have been found to have an underlying predisposing genetic mutation, the most common of which results in cystinuria [2–4]. Cystinuria is an autosomal recessive condition typically diagnosed in adolescence and accounts for 6%–8% of paediatric urolithiasis and 1% of adult cases [5, 6]. Cystinuria is a rare diagnosis in adults and is often not considered. The diagnosis is therefore often missed or delayed and can potentially lead to irreversible renal damage and occasionally necessitate a nephrectomy [7]. This report underscores the need for ongoing vigilance in adult patients presenting with recurrent or resistant urolithiasis for undiagnosed cystinuria.

## 2. Case Report

A 67‐year‐old man presented to the Emergency Department with acute left flank pain and vomiting. Abdominal examination revealed left flank tenderness, and initial urinalysis was positive for both blood and protein. Laboratory investigations (Table 1) revealed acute kidney injury, with serum creatinine rising to 175 *μ*mol/L from a baseline of 100 *μ*mol/L. C‐reactive protein was markedly elevated at 59 mg/L, and reactive thrombocytopenia was demonstrated, with platelets returning to normal (182 × 10^9^/L) after the acute inflammatory event had been managed. Urine cultures showed no significant bacterial growth.

**Table 1:** Laboratory results on admission.

Full blood count		Serum electrolytes		Urinalysis	
Haemoglobin	141 (g/L)	Sodium	138 (mmol/L)	pH	7.5
Haematocrit	0.413 (L/L)	Potassium	4.2 (mmol/L)	Leucocytes	Negative
White cell count	8.61 (×10^9^/L)	Chloride	100 (mmol/L)	Nitrites	Negative
Neutrophils	6.16 (×10^9^/L)	Phosphate	0.85 (mmol/L)	Protein	2+
Platelets	105 (×10^9^/L)	Adjusted calcium	2.59 (mmol/L)	Glucose	Negative
		Uric acid	298 (*μ*mol/L)	Ketones	Negative
C‐reactive protein	59 (mg/L)	Urea	7.5 (mmol/L)	Blood	2+
		Creatinine	175 (*μ*mol/L)		
		eGFR	34 (mL/min)		

**Figure 1:**
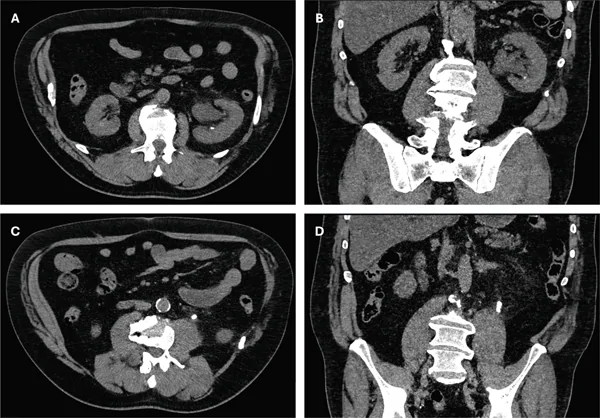
Noncontrast CT urinary tract identifying small left renal stones measuring between 2.6 and 5.0 mm and left‐sided proximal ureteric stones measuring up to 9.5 mm. Average stone density calculated as 650 Hounsfield Units. No right‐sided renal or ureteric stones identified. (A) Axial views of both kidneys with left renal calculi and evident hydronephrosis. (B) Coronal views of both kidneys, showing left renal calculus and hydronephrosis. (C) Axial view of proximal ureters, with left ureteric calculus present. (D) Coronal view identifying multiple proximal left ureteric calculi. *CT protocol details: noncontrast; helical scan mode; slice thickness 0.625 mm; 57 mAs; 120 kVp.*

Noncontrast CT imaging of the urinary tract identified left renal calculi and multiple proximal ureteric stones, measuring up to 9.5 mm in diameter, accompanied by hydronephrosis (Figure 1). Average stone density was calculated as 650 Hounsfield units. Radiological imaging provided no evidence of right‐sided renal or ureteric calculi. The patient underwent emergency rigid cystoscopy and placement of a left JJ stent to decompress the urinary system. He was discharged the following day with plans for definitive stone surgery at a later date.

The patient returned to theatre a few weeks later for left ureteroscopy and laser lithotripsy with exchange of the JJ stent. Intraoperatively, the stent was identified to have mild encrustation and was removed. Semirigid ureteroscopy revealed numerous calculi in the proximal ureter, which were washed into the renal pelvis. Laser lithotripsy was then performed using a flexible ureteroscope and 272‐*μ*m laser fibre; the initial laser settings used were a pulse energy of 0.5 J with a frequency of 10–20 Hz to try and dust the stones. However, very hard, resistant stones were noted during dusting; therefore, the remaining calculi were fragmented with a pulse energy of 1 J and a frequency of 10 Hz. After 24 minutes of laser time, the remaining calculi were deemed dense and resistant to laser fragmentation. During stone extraction, the operating surgeon noted a distinctive sulphurous, “rotten egg” odour, which is characteristic but not diagnostic of cystine stones, raising the suspicion for underlying cystinuria. Extracted calculi were sent for laboratory stone analysis as gold standard investigation. Due to poor visibility from nonevacuated stone dust, a decision was made to re‐insert a JJ stent and abandon the procedure.

A further diagnostic flexible ureteroscopy was performed in the following weeks, which again demonstrated encrustation at the distal end of the JJ stent. No additional calculi were identified, and the patient was rendered stent‐free.

Stone analysis confirmed that the left renal calculi were composed entirely of cystine, establishing the diagnosis of cystinuria. Follow‐up 24‐h urine analysis further confirmed the diagnosis of cystinuria, with increased 24‐h urinary excretion of cystine, ornithine, lysine and arginine when compared to creatinine excretion (Table 2). Initial adjusted serum calcium was borderline elevated at 2.59 mmol/L; however, further investigation revealed normal parathyroid hormone level of 54 pg/mL and repeat adjusted serum calcium of 2.48 mmol/L, excluding hyperparathyroidism. Uric acid levels were also considered normal at 298 *μ*mol/L.

**Table 2:** 24‐h urine analysis divided into day and night samples.

Urine analysis	Daytime urine sample	Nighttime urine sample
Volume (mL)	703	902
Urine creatinine (3.5–24.6 mmol/L)	12.7	8.8
Urine cystine (*μ*mol/L)	1330	1415
Cystine:creatinine ratio (6–34 *μ*mol/mmol cr)	105	161
Urine ornithine (*μ*mol/L)	844	936
Ornithine:creatinine ratio (< 5 *μ*mol/mmol cr)	66	106
Urine lysine (*μ*mol/L)	3751	3298
Lysine:creatinine ratio (7–58 *μ*mol/mmol cr)	295	375
Urine arginine (*μ*mol/L)	716	926
Arginine:creatinine ratio (< 5 *μ*mol/mmol cr)	56	105

Unexpectedly, for cases of cystinuria, the patient′s initial urine pH on admission was alkalotic at 7.5. However, this may have been influenced by an underlying urinary tract infection, despite negative urine cultures, or a laboratory processing error. Repeated urinary pH during his clinical course confirmed acidic urine with pH 6.0, and therefore they were commenced on potassium citrate for medical urinary alkalinisation.

On further discussion, the patient reported a previous stone event at age 18 with spontaneous passage but had remained stone‐free for nearly 50 years. There was no prior diagnosis of cystinuria, and they reported no known family history of urolithiasis. This identified a 49‐year diagnostic delay between initial stone presentation and a diagnosis of cystinuria, highlighting that cystinuria can remain clinically indolent and undiagnosed for decades. A high index of suspicion must be maintained in patients with reported history of stone passage.

Given the high risk of recurrent urolithiasis, the patient was advised to maintain a fluid intake of 4 L per day, and a follow‐up noncontrast CT urinary tract was arranged for surveillance.

## 3. Discussion

Cystinuria is a genetic disorder resulting from mutations in the SLC3A1 and SLC7A9 genes, which encode subunits of the cystine transporter in the proximal renal tubule [8, 9]. These genetic defects impair the reabsorption of cystine, ornithine, lysine and arginine, leading to increased urinary excretion of cystine. Due to the low solubility of cystine at physiological urinary pH, elevated urinary cystine concentrations promote precipitation and subsequent stone formation, manifesting as recurrent urolithiasis. The estimated prevalence of cystinuria is approximately 1 in 7000 individuals [9].

Over 240 different mutations have been identified in SLC3A1 and SLC7A9 resulting in cystinuria [7]. Mutations in SLC3A1 (type A) are inherited in a true autosomal recessive manner requiring two copies of the gene for cystinuria to develop [7]. In contrast, mutations in SLC7A9 (type B) may be inherited in an autosomal dominant manner with incomplete penetrance [7]. Mutations in both SLC3A1 and SLC7A9 (type AB) can also occur in extremely rare cases and present with a mild phenotype [10]. Cases of incomplete penetrance or those with a mild phenotype may present with lower levels of cystine excretion, therefore escaping early detection and remaining undiagnosed [10]. Delay in diagnosis can result in undetected renal calculi, irreversible renal damage and, occasionally, the need for nephrectomy, potentially worsening clinical outcomes compared to those detected early [7].

The majority of patients with cystinuria experience their first stone event in their teenage years [5, 9, 11, 12]. For example, a large multicentre French study of 442 patients reported a median age of first stone presentation of 18.5 years, with most diagnoses occurring between ages 16 and 40 [5]. Similarly, a UK study analysing clinical and genetic data found a median age of first stone event of 24 years, with only 21% of patients presenting after the age of 40 [8]. However, our case identifies that patients may remain undiagnosed for many decades, and cystinuria should be considered at any age, particularly in patients with recurrent or resistant urolithiasis or a positive family history.

Studies indicate that male patients are more severely affected by cystinuria than females [12]. On average, spontaneous passage of renal calculi occurs every 3 years in males, compared to every 5 years in females [12]. When both surgical intervention and spontaneous passage are considered, male patients experience approximately 0.42 stone events per year, whereas female patients have 0.21 events per year [12].

A diagnosis of cystinuria is typically established via renal stone analysis using infrared spectroscopy or X‐ray diffraction or via 24‐h urine analysis [9], both of which occurred in our case. In our centre, 24‐h urine samples are collected over two consecutive 12‐h periods. A container is provided for daytime collection (between 08:00 and 20:00) and a separate container for nighttime collection (between 20:00 and 08:00). Patients are instructed to void upon waking and discard this sample, prior to collecting all subsequent urine in the provided containers. The samples are then returned to the laboratory for processing, where concentrations of excreted dibasic amino acids are expressed relative to creatinine. In cases of cystinuria, 24‐h urine analysis demonstrates elevated levels of cystine, ornithine, lysine and arginine relative to creatinine, reflecting impaired renal reabsorption. Twenty‐four‐hour urine analysis can also be used to monitor ongoing disease activity in response to medical management [7].

Genetic testing for cystinuria may be performed in specialist centres in cases of atypical presentation, uncertain inheritance or for genetic counselling [9]. Genetic analysis is performed via direct Spanger sequencing, polymerase chain reaction and next‐generation sequencing–based panel analysis to identify genetic variations in SLC3A1 and SLC7A9 [9]. However, genetic analysis should be consistent with clinical and biochemical results and is not considered mandatory for a diagnosis [9]. Given the autosomal recessive nature of many cases of cystinuria, siblings of confirmed cases should be offered investigation as they may remain asymptomatic and undetected. Sodium cyanidenitroprusside is often used as a screening test with sensitivity quoted as 72% and specificity 95% [7].

Analysis of renal calculi may reveal hexagonal cystine crystals which are highly specific for cystinuria [9, 13]. Most stones are composed entirely of cystine, although mixed composition can occur. Cystine calculi are characteristically pink or yellow, turning green upon surgical removal and exposure to air [13]. These stones are frequently resistant to extracorporeal shock wave lithotripsy, making ureteroscopy or percutaneous nephrolithotomy (PCNL) the preferred treatment modalities [9, 13]. Due to the frequency of required procedures, ureteroscopy is generally favoured to minimise the need for recurrent PCNL. Patients with cystinuria also tend to have longer recovery times [11], underscoring the importance of preventive management. Optimal care involves regular surveillance imaging, ongoing follow‐up with Urology and implementation of preventive strategies in collaboration with Nephrology and Dietetics. Inadequate follow‐up and management can increase the risk of renal impairment.

Patients are encouraged to have a fluid intake of 3.5–4 L/day to produce 3 L of urine output, reducing the urine cystine concentration and crystal precipitation [11, 13]. Increased fluid intake should be consumed prior to bedtime and on wakening [9]. Additional lifestyle advice includes adapting a low sodium diet as low urinary sodium excretion reduces cystine excretion, due to coupled transport in the renal tubule [14]. A vegetarian diet may be suggested after adequate clinical counselling as low animal protein intake with high intake of fruit and vegetables increases the urinary pH and prevents cystine stone formation [15].

Medical management options include alkalising the urine with potassium citrate or sodium bicarbonate to reach an optimal urinary pH of 7.5–8.0 [9, 16]. Potassium citrate is the preferred modality as it avoids a high sodium load [16]. Sodium bicarbonate however can be utilised in cases of severe renal insufficiency or intolerance to potassium citrate [9]. Resistant cases may be offered chelation with tiopronin or D‐penicillamine which cleave cystine to form mixed disulfide complexes [9]. These complexes are significantly more soluble than cystine and therefore effectively reduce urine cystine levels. However, adverse effects have been documented including immune complex membranous glomerulopathy, mucocutaneous lesions and haematological reactions [9].

Although this case highlights that cystinuria should be considered in patients of any age, several limitations need to be addressed. As the findings presented are based on a single patient, they may not be generalisable to the wider population. Additionally, the retrospective nature of the case with limited follow‐up provides little insight into the long‐term consequences of undiagnosed cystinuria. Genetic confirmation was also not performed, and the exact method of stone analysis was not documented. Future research should focus on the long‐term consequences of clinically indolent and undiagnosed cystinuria.

This case demonstrates that cystinuria, although typically diagnosed in childhood or adolescence, can be diagnosed in older adults after a period of clinical indolence. Clinicians should therefore maintain a high index of suspicion for cystinuria in adult patients with unexplained or refractory stone disease. Optimal management centres on preventing recurrent stone formation through aggressive fluid intake, dietary modifications and medical alkalinisation of urine, as cystine calculi are often resistant to surgical intervention. Multidisciplinary follow‐up with Urology, Nephrology and Dietetics is important to reduce the risk of renal impairment.

## Funding

No funding was received for this manuscript.

## Ethics Statement

Written informed consent was obtained from the individual patient involved in this case report for clinical details of their presentation, management, clinical course and accompanying imaging to be published. All reasonable efforts have been made to protect patient confidentiality, and all identifying information has been anonymised.

## Conflicts of Interest

The authors declare no conflicts of interest.

## Data Availability

The data that support the findings of this study are available from the corresponding author upon reasonable request.
